# Cell-mediated Protection in Influenza Infection

**DOI:** 10.3201/eid1201.051237

**Published:** 2006-01

**Authors:** Paul G. Thomas, Rachael Keating, Diane J. Hulse-Post, Peter C. Doherty

**Affiliations:** * St. Jude Children's Research Hospital, Memphis, Tennessee, USA

**Keywords:** influenza, cell-mediated immunity, vaccine, highly pathogenic virus, perspective

## Abstract

Current vaccine strategies against influenza focus on generating robust antibody responses. Because of the high degree of antigenic drift among circulating influenza strains over the course of a year, vaccine strains must be reformulated specifically for each influenza season. The time delay from isolating the pandemic strain to large-scale vaccine production would be detrimental in a pandemic situation. A vaccine approach based on cell-mediated immunity that avoids some of these drawbacks is discussed here. Specifically, cell-mediated responses typically focus on peptides from internal influenza proteins, which are far less susceptible to antigenic variation. We review the literature on the role of CD4+ and CD8+ T cell–mediated immunity in influenza infection and the available data on the role of these responses in protection from highly pathogenic influenza infection. We discuss the advantages of developing a vaccine based on cell-mediated immune responses toward highly pathogenic influenza virus and potential problems arising from immune pressure.

## Abstract 2

Cell-mediated immune responses should be considered in vaccination protocols.

Vaccine approaches against respiratory virus infections such as influenza have relied on inducing antibodies that protect against viral infection by neutralizing virions or blocking the virus's entry into cells. These humoral immune responses target external viral coat proteins that are conserved for a given strain. Antibody-mediated protection is therefore effective against homologous viral strains but inadequate against heterologous strains with serologically distinct coat proteins. This distinction is of consequence since many viruses rapidly mutate their coat proteins; an effective humoral response–based vaccine against a form of the virus may be ineffective against next season's variant. In contrast, T cells, which mediate cellular immune responses, can target internal proteins common to heterologous viral strains. This property gives vaccines that induce protective cellular immune responses the potential to protect against heterologous viral strains.

Antigen-specific ligation of T-cell receptors induces effector mechanisms that either directly or indirectly promote lysis of infected cells. Functionally distinct T-cell subsets are broadly identified according to their differential expression of CD4 and CD8 coreceptors. The CD4+ T helper cells are primarily responsible for helping other immune cells through direct cell-cell interactions or by secreting cytokines after recognizing viral peptides bound to major histocompatibility complex (MHC) class II molecules. The cytotoxic T lymphocytes (CTLs) typically express CD8 and induce apoptosis of cells on which they recognize foreign antigens presented by MHC class I molecules, providing a defense against intracellular pathogens such as viruses. This association of phenotype and function is not absolute, since CD4+ cells may exhibit lytic activity, while CD8+ cells secrete antiviral cytokines, notably interferon-γ (IFN-γ) and tumor necrosis factor. Greater understanding of how each subset contributes to protective immunity and how T-cell memory is maintained and recalled in a secondary infection would contribute to development of effective vaccines that use these basic features of the immune response.

## Immune Models of Influenza

Influenza is a contagious, acute respiratory disease caused by infection of the host respiratory tract mucosa by an influenza virus (*1*). The influenza A viruses infect host epithelial cells by attaching to a cellular receptor (sialic acid) by the viral surface protein hemagglutinin (HA). The virus is subsequently released because of the action of another surface glycoprotein, the enzyme neuraminidase (NA), several hours after infection.

Mouse models of influenza A virus pneumonia provide a well-developed experimental system to analyze T cell–mediated immunity. In particular, the T-cell immune response to influenza infection has been well characterized in C57BL/6 (B6,H2^b^) mice. While influenza infection of mice does not precisely replicate the natural infection in human, avian, or other vertebrate species, the availability of reagents and genetically modified mouse models has enabled extensive analysis of the cellular immune response. Emerging evidence indicates that findings from mouse studies are pertinent to immunopathology in human disease. In the BL/6 model, virus is cleared 10 days after infection, with no indication of persistent antigen or viral RNA (*2*). Recovery or prevention of influenza relies on targeting both innate and adaptive responses to the respiratory tract mucosa.

## CD8+ T-cell Response to Influenza

Much of the current knowledge on murine CD8+ T-cell responses to influenza has come from analyzing the response to challenge with the HKx31 (H3N2) and PR/8 (H1N1) influenza viruses. A role for CD8+ T cells in protective immunity has been discerned from studies citing delayed influenza virus clearance in CD8+ T cell–deficient mice (*3**,**4*). Furthermore, CD8+ T cells can promote recovery from otherwise lethal secondary viral infections in mice that lack mature B cells or antibodies (*5**,**6*), and cloned influenza-specific CTLs can passively transfer protection (*7*). Despite a seemingly protective role for CD8+ T cells, vaccination with dominant influenza determinants in either a vector or in a recombinant form is only mildly protective (*8**–**10*). Moreover, in a T cell–receptor transgenic mouse model, devoid of antibodies, influenza-specific CTL can either contribute to survival or exacerbate lethal influenza pneumonia (*11*). This study highlights the need to understand the many facets of the immune response to influenza.

The influenza A virus–specific CD8+ T-cell response has been characterized by using intracellular cytokine staining and MHC class I tetramer labeling. These techniques have enabled each phase of the response to be tracked. After intranasal infection, priming, activation, and expansion of naive influenza-specific CD8+ T cells occur in the draining mediastinal lymph node 3–4 days after infection (*12**,**13*). The antiviral capacity of these virus-specific CD8+ cells is strongly dependent on their ability to migrate and localize to the lungs and infected airway epithelium (*14*), where they appear 5–7 days after infection (*15*). Because viral replication is confined to cells in the respiratory epithelium (*16**,**17*), CD8+ T cells exert their effector functions at this site, producing antiviral cytokines and lysing target cells presenting viral determinants for which they bear a specific T-cell receptor. Lysis of infected epithelial cells is mediated by exocytosis granules containing perforin and granzymes (*18**,**19*). The release of perforin and granzymes from influenza-specific CTLs is tightly regulated, occurring shortly after activation at or near the contact point between CTLs and the infected target cell (*18*).

Influenza-specific CD8+ T cells recognize multiple viral epitopes on target cells and antigen-presenting cells. The HKx31 and PR8 strains share 6 internal genes derived from PR8 that are processed to generate peptides recognized by influenza-specific CD8+ T cells. The primary response to either strain is dominated by CD8+ T cells' recognition of 2 determinants, the nucleoprotein (NP_366-374_, H2D^b^) (*20*) and the acid polymerase (PA_224-233_, H2D^b^) (*21*). A similarly low proportion of CD8+ T cells recognizes 4 other determinants: the basic polymerase subunit 1 (PB1_703-711_, H2K^b^), nonstructural protein 2 (NS2_114-121_, H2K^d^), matrix protein 1 (M1_128-135_, H2K^b^), and a protein derived from an alternative open reading frame within the PB1 gene (PB1-F2_62-70_, H2D^b^) (*22*). The subsequent memory populations appears to be stable; D^b^NP_366-374_ and D^b^PA_224-233_ CD8+ memory cells are still detectable >570 days after initial infection (K. Kedzierska and J. Stambas, unpub. data).

Secondary influenza-specific CTL responses arise ≈2 days faster than the primary response, with a greatly increased level of activity. Depletion of CD8+ T cells reduces the capacity of primed mice to respond to influenza infection, which indicates a role for CD8+ T cells in the protective secondary response. Prime and challenge experiments can be conducted with HKx31 and PR/8 as all of the recognized epitopes are derived from internal proteins. Furthermore, cross-reactive neutralizing antibodies are avoided because HKx31 and PR/8 express different surface HA and NA or proteins. Despite a similar magnitude to D^b^PA_224-233_ in the primary response, D^b^NP_366-374_-specific CD8+ T cells dominate the secondary response to HKx31→PR/8 challenge, accounting for up to 80% of the influenza-specific CD8+ T cells. This dominance is maintained in the memory population; the numbers of NP-specific CD8+ T cells exceed all other quantified influenza-specific CD8+ T-cell populations (*23*). Despite the NP dominance, CD8+ T cells specific for the other 5 determinants can still be isolated after secondary challenge, albeit at low frequency.

Conservation of these 6 internal genes and persistence of the corresponding antigen-specific CD8+ T cells makes these genes an attractive target for vaccine therapies. However, although cell-mediated immunity can promote viral clearance, it does not provide sterile resistance because, unlike humoral immunity, it cannot prevent infection of the host cells. In humans, the level of influenza-specific CTLs correlates with the rate of viral clearance but not with susceptibility to infection or subsequent illness (*24*). Despite this limitation, vaccines that promote cell-mediated immunity may be a favorable option to fight potentially lethal, highly pathogenic influenza strains.

## CD4+ T cell–specific Responses to Viruses

In contrast to the body of literature that has characterized the role of CD8+ T cells specifically in models of influenza infection, relatively little is known about the role of CD4+ T cells as direct mediators of effector function. That CD4+ T-cell help is central to adaptive immunity is well established, but few antigen-specific systems have been developed to dissect the role of CD4+ T cells in a viral infection. While knowledge of CD8+ T-cell antigen-specific responses has increased substantially in the past several years as a result of tetramer technology, these reagents have been more difficult to develop for the CD4+ subset. Further, identification of CD4+ T cell–specific epitopes has been less successful for a variety of pathogens. For instance, in influenza, the CD8+ restricted epitopes have all been largely identified for some time, particularly in the BL/6 model system; in contrast, only very recently have confirmed CD4 epitopes been found, and they are much more poorly characterized (*25*).

Still, a substantial amount of work has been done with various knockout, depletion, and cell-transfer models to investigate the role of CD4+ T cells in primary, secondary, and memory responses to influenza infection in the mouse model (*26**,**27*). Controversy still exists in the field, and an antigen-specific system would help address it, but certain findings appear to be consistent across different experimental systems (*28*).

In the primary response, CD4+ T cells are not required for expansion or development of functional CD8+ CTL (*27**,**29*), which may in part result from the ability of influenza virus to directly activate dendritic cells, aiding in the development of CD8+ responses that substitute for functional CD4+ T cells (*30*). Similarly, in the case of a murine γ-herpesvirus, the lack of CD4+ T cells can be compensated for by the addition of anti-CD40 stimulation (*31*). In mice in which both the CD4+ T-cell and B-cell compartments were defective, the primary CD8+ T-cell response to influenza appeared to be stunted in terms of recruitment and expansion (vs. mice in which B cells alone were knocked out); the remaining CD8+ T cells had a robust level of functionality as assayed by IFN-γ intracellular cytokine production (*27*). The defect in the CD8+ T-cell primary response was less obvious in mice with intact B cells, though viral clearance was delayed. Still, not until the secondary and memory responses are examined can the dramatic effect of CD4+ T-cell deletion be observed.

In multiple systems, a defect of CD8+ T-cell secondary and memory responses have been observed when the primary response lacks CD4+ T cells (*26**,**32**,**33*). In influenza, a dramatic drop was observed in the size and magnitude of the recall response to secondary infection. The rate of viral clearance was also slowed considerably, beyond the degree seen in the primary response. Similarly, in the *Listeria monocytogenes* model system, the primary response was largely intact, while the long-term memory response was defective (*34*). In mice that lacked CD4+ T cells during the primary response, the memory pool of CD8+ T cells was initially similar in size and functionality to that seen in wild-type mice but began to decline after longer intervals, leading eventually to the recrudescence of the infection. Secondary challenge also demonstrated a reduced antigen-specific CD8+ T-cell compartment.

In the influenza model, although the draining lymph node and spleen CD8+ responses were defective in secondary infection of CD4+ T cell–deficient mice, the CD8+ T-cell responses in bronchoalveolar lavage were equivalent to those seen in wild-type mice (*29*). This finding implies that the high levels of activation and inflammation, in large part mediated by innate immune effectors at the site of infection, were capable of providing the right maturation milieu to expand the response to wild-type levels; this finding suggests CD4+ T cell–specific help is not required at the site of the pathologic changes, at least when the infection induces a high level of other immune stimulation, though it is essential in the lymphoid organs in the generation and maintenance of memory.

A role for CD4+ T cells as effectors has been found in a number of other systems, including the mouse γ-herpesvirus model (*35*) and in HIV-infected humans (*36**,**37*). In these studies, CD4+ T cells contribute to infection control by supplementing their helper role with cytotoxicity. In the case of the γ-herpesvirus, the effector CD4+ population was important only in immunoglobulin –/– μMT mice, while the HIV studies were conducted in infected (and presumably immune-irregular) patients. However, effector CD4+ T cells have been found in multiple stages of the disease and in long-term patients whose disease is not progressing because viral replication is controlled. Finally, a recent report demonstrated a similar cytotoxic CD4+ T-cell effector population in protozoan-infected cattle (*38*).

Relatively few established mouse models are available for studying the CD4+ response to influenza virus. On the IA^d^ BALB/c background, an HA epitope was discovered, and a transgenic mouse was developed to analyze specific responses (the HNT model) (*39*). This model has been extremely useful for studying several aspects of CD4+ biology in influenza infection, particularly in regards to aging and the development of primary responses leading to acute memory (*39*). Several investigators have introduced external epitopes in influenza to follow CD4+ T-cell responses in defined systems. These include the hen egg lysozyme p46–63 sequence (*40*) and the ovalbumin 323–339 (OT-II) epitope inserted into the NA stalk of WSN influenza virus (*41*). We have inserted the OT-II epitope into the HA of the PR8 H1N1 virus and the X31 H3N2 virus. In contrast to the robust responses achieved with CD8+ T-cell epitopes and transgenics, the CD4+ T-cell responses seem relatively weak (unpub. data). Other naturally occurring epitopes have similarly low frequencies after infection (*25*). The antigen-specific CD4+ response may not develop the dramatic immunodominance hierarchies that are well-known for CD8+ T cells and may be directed at many epitopes, more than are seen in the more-delimited CD8+ T-cell response. Much work needs to be done before this conclusion is certain, and examples of respiratory infections in mice produce robust and dominant responses toward individual class II epitopes (*42*).

## Cell-mediated Protection against Highly Pathogenic Influenza

Highly pathogenic H5N1 influenza emerged in 1997, followed by several waves of infection from 2002 until now (*43*). The viruses have been remarkably virulent in multiple animal models, including mice, but little work has been done to characterize the protective immune responses toward H5N1 viruses. A series of reports has shown strong protection toward other highly pathogenic viruses mediated by cellular responses, in the absence of neutralizing antibody. Antibody-deficient mice infected with a mild, passaged strain of an H3N2 virus were more likely to survive than naive controls when challenged with a highly pathogenic H3N8 duck virus compared to naive controls (*44*). A double-priming protocol provided increased protection from a lethal H7N7 challenge, which correlated with an increased pool of cross-reactive antigen-specific CD8+ T cells (*45*). In both these cases, the initial phase of infection and viral growth seemed similar to that occurring in immunologically naive mice, but a rapid decrease in viral titers occurs after a few days of infection.

Since the emergence of the H5N1 viruses, concern has arisen that the biological activity of these viruses, including their diverse tissue tropism in a number of animal models, may influence the ability of immune responses to control infection. Furthermore, some pathology associated with these viruses has been attributed to extremely high levels of inflammatory cytokines produced in response to infection, which suggests a negative role for immune responses. However, the few studies that have been performed have shown promising results for the potential of cell-mediated responses to contribute to the control of infections. A prime-challenge protocol using an H9N2 isolate with 98% homology to the internal genes of the A/HongKong/156/97 H5N1 protected against the otherwise lethal challenge (*46*) with a virus with a highly cleavable H5, a characteristic of all the pathogenic H5 viruses. The priming protocol generated significant CTL activity directed at the NP and PB2 proteins.

Our own work has indicated a similar ability of cell-mediated immunity to protect against virulent H5N1 challenge. In a preliminary experiment, we primed mice with the H1N1 PR8 strain and the H3N2 X31 strain followed by a challenge with A/Vietnam/1203/2004, one of the most lethal H5N1 viruses, which causes severe pathologic changes, even in ducks. While 9 of 10 naive mice died, 9 of 10 primed mice survived past day 10 of infectious challenge and recovered substantial weight (Figure). The fact that both groups lost weight indicated protection was occurring by delayed cell-mediated responses, rather than by the "immediate" cross-protective antibody response.

**Figure:**
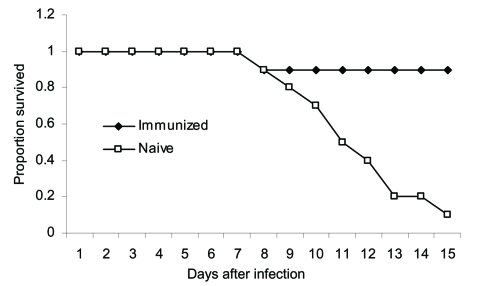
Apparent cell-mediated protection against highly pathogenic H5N1 influenza virus. Mice (10 in each group) were immunized by intraperitoneal injection of PR8, followed by intraperitoneal injection 4 weeks later of X31. Four weeks after the second immunization, immunized or naive mice were infected with 300 mouse lethal dose 50% of A/Vietnam/1203/2004.

## Cell-mediated Vaccine for Highly Pathogenic Influenza?

Despite the systems currently in place for manufacturing and distributing an influenza vaccine, pandemic influenza will require a substantially different approach. The standard influenza vaccine given during the infectious season is made from a reassortant seed strain containing the HA and NA of the circulating virus with the internal genes of a vaccine strain, usually PR8. The seed strain is grown in eggs and is formaldehyde inactivated. This strategy does not prime strong CD8 CTL responses, but it is effective in providing antibody-mediated protection to closely homologous strains (*47*).

One drawback to this approach is the length of time required to develop a seed strain, amplify it, and manufacture it into distributable vaccine. In the case of a potential influenza pandemic, the delivery of vaccine on this schedule would not prevent the spread of the epidemic in many countries. Furthermore, antigenic drift can occur between the original selection of the seed strain and circulating viruses before the vaccine is ready for distribution (*48*). This problem was faced recently in a nonpandemic situation in 2003 and 2004 when the circulating Fujian strain of H3N2 influenza had drifted from the vaccine strain (*49*). While the Fujian strain was predicted to be circulating at the time of vaccine delivery, a recombined seed strain could not be isolated in time for vaccine production. Although the ensuing influenza season was not as severe as initially feared, the situation highlighted a problem with the current vaccine strategy. Evidence of antigenic drift is already evident in the most recent outbreaks of H5N1 (*48*).

Several groups have developed reverse genetics–based methods that could speed the production of seed viruses as well as proposals for growing viruses in cell culture rather than in embyronated chicken eggs, which would allow for a much faster scale up in response to an epidemic (*50*). These technologies have not been approved yet for human use, though trials are underway.

Even if the development of recombinant seed strains by reverse genetics becomes standard over the next few years, questions remain about how effective the current formaldehyde-inactivated seed strain strategies would be against pandemic strains, particularly the currently circulating H5N1 strains. Assuming that seed strains could be produced rapidly, several weeks would be required to manufacture a relevant number of doses of vaccine. To address this concern, several governments have been stockpiling vaccines based on H5N1 viruses that have been circulating over the last few years. While these vaccines may provide some protection, substantial evolution and antigenic drift seem to be occurring, rendering the stockpiled strains less and less useful (*48*).

An approach based on conserved cellular epitopes within the internal genes has the advantage of subverting all of these issues. While cellular immunity is not sterilizing, it prevents illness and death in animal models (*3*). Common and immunodominant epitopes among circulating nonavian strains have been identified, and many of the same models and algorithms can be used to make predictions against the pathogenic strains (*51*). Mouse models are now available that have human leukocyte antigen (HLA) alleles, and they appear to recapitulate human epitope use. As described earlier, protection against death from highly pathogenic viruses has been shown in multiple systems. Cross-protective cell-mediated immunity has been found in birds for circulating chicken H5N1 and H9N2, both of which have been suggested as potential human pandemic strains (*52*). The notion of a "universal" vaccine for highly pathogenic strains is attractive.

Antigenic drift due to immunologic pressure is also a concern with a CD8- or CD4-based vaccine approach. Reports have suggested that CD8+ epitopes under pressure will mutate to escape protective immunity (*11*). The mutation of an NP epitope that binds HLA-B35 present in strains of virus from the 1930s through the present indicates that even in nonpandemic years, immunologic pressure from cross-protective CD8+ T cells is enough to drive the evolution of the virus (*53*). In contrast, though, other dominant epitopes do not appear to be under the same pressure (*54*).

Several human peptide epitopes that have been described and characterized show evidence of remarkably little mutation over many generations of viral evolution. In the most recent outbreaks of H5N1 virus, some of these peptides are conserved in viruses isolated from human patients (Table). The conservation of so many peptides from such distantly related viruses suggests that they may be less susceptible to antigenic drift than the HA and NA glycoproteins. Vaccines that promote strong memory CTL activity toward these peptides and MHC, in combination with the antibody-based approaches already underway, could help prevent pandemic influenza. This approach could potentiate immunologic pressure on the vaccine-targeted epitopes, but an immunization strategy that targets a large number of epitopes along with the natural restriction on epitope structure due to viral function should mitigate this effect. Some evidence shows that highly conserved CTL epitopes are restricted from mutation by viral structural requirements. Given the large number of influenza viruses sequenced over time, we should be able to make reasonable assumptions about the identity of these epitopes in MHC-diverse populations and focus on how to facilitate the development of strong immune responses toward them.

**Table:** Conservation of human NP and M1 epitopes between H1N1 PR8 and 3 human isolates of H5N1 viruses (A/Hong Kong/156/1997, A/Hong Kong/213/2003, and A/Vietnam/1203/2004)*

Epitope	HLA restriction	PR8 sequence	Conservation
NP 383–391	B*2705	SRYWAIRTR	3/3 identical
NP 418–426	B*3501	LPFDRTTIM	0/3 identical
NP 44–52	A*01	CTELKLSDY	2/3 identical (156 Y9Q)
NP 265–273	A*03	ILRGSVAHK	3/3 identical
NP 188–198	A*1101	TMVMELVRMIK	3/3 V7I mutation
NP 380–388	B*08	ELRSRYWAI	3/3 identical
NP 174–184	B*2705	RRSGAAGAAVK	2/3 identical (156 V10I)
M1 58–66	A*0201	GILGFVFTL	3/3 identical
M1 27–35	A*03	RLEDVFAGK	2/3 mutated (1203, 213 both R1K)
M1 13–21	A*1101	SIIPSGPLK	3/3 identical

*All 3 isolates were compared to the mouse-adapted PR8 strain and differences are reported. Sequences obtained from the Influenza Sequence Database (*55*). NP, nucleoprotein; HLA, human leukocyte antigen.

## Appendix Bibliography

Further literature support for the material discussed in this article is available.

1. Arnold PY, Vignali KM, Miller TB, La Gruta NL, Cauley LS, Haynes L, et al. Reliable generation and use of MHC class II:gamma2aFc multimers for the identification of antigen-specific CD4(+) T cells. J Immunol Methods. 2002;271:137–51.
2. Belz GT, Xie W, Doherty PC. Diversity of epitope and cytokine profiles for primary and secondary influenza a virus-specific CD8+ T cell responses. J Immunol. 2001;166:4627–33.
3. Boon AC, de Mutsert G, Fouchier RA, Sintnicolaas K, Osterhaus AD, Rimmelzwaan GF. Preferential HLA usage in the influenza virus-specific CTL response. J Immunol. 2004;172:4435–43.
4. Cerwenka A, Morgan TM, Harmsen AG, Dutton RW. Migration kinetics and final destination of type 1 and type 2 CD8 effector cells predict protection against pulmonary virus infection. J Exp Med. 1999;189:423–34.
5. Chen W, Bennink JR, Morton PA, Yewdell JW. Mice deficient in perforin, CD4+ T cells, or CD28-mediated signaling maintain the typical immunodominance hierarchies of CD8+ T-cell responses to influenza virus. J Virol. 2002;76:10332–7.
6. Doherty PC, Topham DJ, Tripp RA, Cardin RD, Brooks JW, Stevenson PG. Effector CD4+ and CD8+ T-cell mechanisms in the control of respiratory virus infections. Immunol Rev. 1997;159:105–17.
7. Falk K, Rotzschke O, Deres K, Metzger J, Jung G, Rammensee HG. Identification of naturally processed viral nonapeptides allows their quantification in infected cells and suggests an allele-specific T cell epitope forecast. J Exp Med. 1991;174:425–34.
8. Flynn KJ, Belz GT, Altman JD, Ahmed R, Woodland DL, Doherty PC. Virus-specific CD8+ T cells in primary and secondary influenza pneumonia. Immunity. 1998;8:683–91.
9. Flynn KJ, Riberdy JM, Christensen JP, Altman JD, Doherty PC. In vivo proliferation of naive and memory influenza-specific CD8(+) T cells. Proc Natl Acad Sci U S A. 1999;96:8597–602.
10. Hou S, Hyland L, Ryan KW, Portner A, Doherty PC. Virus-specific CD8+ T-cell memory determined by clonal burst size. Nature. 1994;369:652–4.
11. Hu N, D'Souza C, Cheung H, Lang H, Cheuk E, Chamberlain JW. Highly conserved pattern of recognition of influenza A wild-type and variant CD8(+) CTL epitopes in HLA-A2(+) humans and transgenic HLA-A2(+)/H2 class I-deficient mice. Vaccine. 2005;23:5231–44.
12. Kilbourne ED. Future influenza vaccines and the use of genetic recombinants. Bull World Health Organ. 1969;41:643–5.
13. Lamb RA, Krug RM. Orthomyxoviridae: the viruses and their replication. In: Fields BN, Knipe DM, Howley PM, Chanock RM, Melnick JL, Monath TP, et al., editors. Fields virology. 3rd ed. Philadelphia: Lippincott-Raven Publishers; 1996. 1353–95.
14. Lawson CM, Bennink JR, Restifo NP, Yewdell JW, Murphy BR. Primary pulmonary cytotoxic T lymphocytes induced by immunization with a vaccinia virus recombinant expressing influenza A virus nucleoprotein peptide do not protect mice against challenge. J Virol. 1994;68:3505–11.
15. Lund JM, Alexopoulou L, Sato A, Karow M, Adams NC, Gale NW, et al. Recognition of single-stranded RNA viruses by Toll-like receptor 7. Proc Natl Acad Sci U S A. 2004;101:5598–603.
16. Rott R, Klenk HD, Nagai Y, Tashiro M. Influenza viruses, cell enzymes, and pathogenicity. Am J Respir Crit Care Med. 1995;152:S16–9.
17. Stephenson I, Nicholson KG, Wood JM, Zambon MC, Katz JM. Confronting the avian influenza threat: vaccine development for a potential pandemic. Lancet Infect Dis. 2004;4:499–509.
18. Topham DJ, Doherty PC. Clearance of an influenza A virus by CD4+ T cells is inefficient in the absence of B cells. J Virol. 1998;72:882–5.
19. Wood JM, Robertson JS. From lethal virus to life-saving vaccine: developing inactivated vaccines for pandemic influenza. Nat Rev Microbiol. 2004;2:842–7.
20. Wraith DC, Vessey AE, Askonas BA. Purified influenza virus nucleoprotein protects mice from lethal infection. J Gen Virol. 1987;68:433–40.
21. Zaunders JJ, Dyer WB, Wang B, Munier ML, Miranda-Saksena M, Newton R, et al. Identification of circulating antigen-specific CD4+ T lymphocytes with a CCR5+, cytotoxic phenotype in an HIV-1 long-term nonprogressor and in CMV infection. Blood. 2004;103:2238–47.
